# Time–frequency analysis of gustatory event related potentials (gERP) in taste disorders

**DOI:** 10.1038/s41598-024-52986-5

**Published:** 2024-01-30

**Authors:** Mariano Mastinu, Lisa Sophie Grzeschuchna, Coralie Mignot, Cagdas Guducu, Vasyl Bogdanov, Thomas Hummel

**Affiliations:** 1 Department of Otorhinolaryngology, Smell & Taste Clinic, Technische Universität Dresden, Dresden, Germany; 2 Dokuz Eylül University Faculty of Medicine Department of Biophysics, 35320 Balçova, Izmir, Turkey

**Keywords:** Sensory processing, Gustatory cortex

## Abstract

In taste disorders, the key to a correct diagnosis and an adequate treatment is an objective assessment. Compared to psychophysical tests, EEG-derived gustatory event-related potentials (gERP) could be used as a less biased measure. However, the responses identified using conventional time-domain averaging show a low signal-to-noise ratio. This study included 44 patients with dysgeusia and 59 healthy participants, who underwent a comprehensive clinical examination of gustatory function. gERPs were recorded in response to stimulation with two concentrations of salty solutions, which were applied with a high precision gustometer. Group differences were examined using gERP analyzed in the canonical time domain and with Time–Frequency Analyses (TFA). Dysgeusic patients showed significantly lower scores for gustatory chemical and electrical stimuli. gERPs failed to show significant differences in amplitudes or latencies between groups. However, TFA showed that gustatory activations were characterized by a stronger power in controls than in patients in the low frequencies (0.1–4 Hz), and a higher desynchronization in the alpha-band (8–12 Hz). Hence, gERPs reflect the altered taste sensation in patients with dysgeusia. TFA appears to enhance the signal-to-noise ratio commonly present when using conventional time-domain averaging, and might be of assistance for the diagnosis of dysgeusia.

## Introduction

According to the German Working Group on Olfactology and Gustology, around 50,000 people worldwide are affected by smell and taste dysfunction each year^1^. Since smell and taste disorders are generally not life threatening, they are often overlooked by physicians and sometimes by the patients themselves. Often, however, they severely reduce the patients’ quality of life due to diminished enjoyment of foods and lack of an important warning sensor^2,3^. Anxiety disorders or the development of depression are therefore not uncommon for the patients with disturbance of the chemical senses^4,5^.

The largest number of currently existing gustatory procedures for evaluating the gustatory sensitivity with proven reliability are based on psychophysical methods^6–9^. They allow quantitative assessment of taste function. However, the most significant taste disorders are qualitative^10^. The diagnosis depends on the patient’s judgement; therefore, the test results can potentially be skewed. Although electrophysiological responses to tastes can be obtained at the human tongue^11–13^, they are limited to the assessment of quantitative taste disorders. Thus, the central processing of gustatory signals and, hence, their disturbance is not covered by these assessments at a peripheral level.

Electroencephalogram (EEG)-based efforts to record the central processing of gustatory signals as electric brain activity in response to electrical or chemical stimulation of gustatory sensors have led to the registration of gustatory event-related potentials (gERP)^14^. The reduced tactile irritation of the tongue through a pulsed application of continuous tasteless spray pulse intermixed with taste stimuli, and the controlled temperature and humidity allow to derive selective gERP^15^. These studies were supported by numerous electro- and magneto-encephalography studies^16–19^.

Singh et al. and others found greater stimulus responses over the right hemisphere of the brain compared to the left hemisphere implying a dominant right-hemispheric taste processing^20,21^. At the same time, the topographical distribution of amplitudes in response to gustatory stimuli suggested that different stimulus qualities activate different areas of the brain^21^.

Still, until now only a few studies have recorded gERP in patients with taste disorders. Kobal (1985) detected reduced amplitudes and prolonged latencies of gERP peaks in patients with reduced taste sensitivity^15^. Furthermore, the lateralized examination of a patient with a unilateral loss of taste showed a regular stimulus response only on the contralateral side. These findings indicate that gERP recordings are a valuable tool to measure human taste function, and it can assess gustatory dysfunction. However, the responses identified using conventional time-domain averaging show a low signal-to-noise ratio^20^. This could be partially due to temporal jitter between trials that are summed using traditional across-trial averaging procedures. Recent studies characterized olfactory EEG responses in the time–frequency domain. While the time domain averaging gives an idea of the phase-locked activity of the brain, the time–frequency analysis (TFA) reflects the non-phase locked activity, meaning that it can reveal synchronization and desynchronization of a neural population related to the event^22–25^.

Recently, the application of TFA to gERPs, which were elicited after presentation of four primary taste qualities (sweet, sour, salty, and bitter) in a group of 16 healthy volunteers, revealed a synchronization in the delta frequency range (1–4 Hz), which was associated with the coding of taste information, immediately after stimulus presentation. Also, desynchronization activity was detected in higher frequency bands (alpha: 8–12 Hz) starting at 0.5 s after stimulus onset^26^.

The goals of the present study were (1) to investigate differences between healthy controls and patients with qualitative dysgeusia in the processing of gustatory stimuli and (2) to examine TFA in terms of the clinical diagnostic evaluation of gERP.

## Results

### Patients characteristics

Healthy controls were younger than patients (F_1,101_ = 12.58, *p* < 0.001). Additionally, the chi-square test revealed that frequency of women was higher in the patients group (χ^2^ = 5.48; *p* = 0.017). Average duration of dysgeusia was 18.5 ± 4.4 months. Patients’ group characteristics can be found as Supplementary Table S1 online. Patients had higher scores in the short form of the Beck Depression Inventory questionnaire (BDI-FS) compared to healthy subjects (patients: 2.34 ± 0.33 points; controls: 0.78 ± 0.29 points; F_1,99_ = 12.56, *p* < 0.001).

### Olfactory testing

Olfactory function, as indicated by the sum of Threshold, Discrimination and Identification test score (TDI), did not differ (*p* > 0.05) between patients with dysgeusia and controls. Similarly, no significant difference was observed between the two groups for self-rated olfactory abilities (*p* > 0.05). Thirty-three patients were classified as normosmic, 5 of the patients had hyposmia and 6 had anosmia, while in the control group 51 subjects were classified as normosmic, whereas 8 of them were diagnosed with hyposmia.

### Gustatory testing

**Figure 1:**
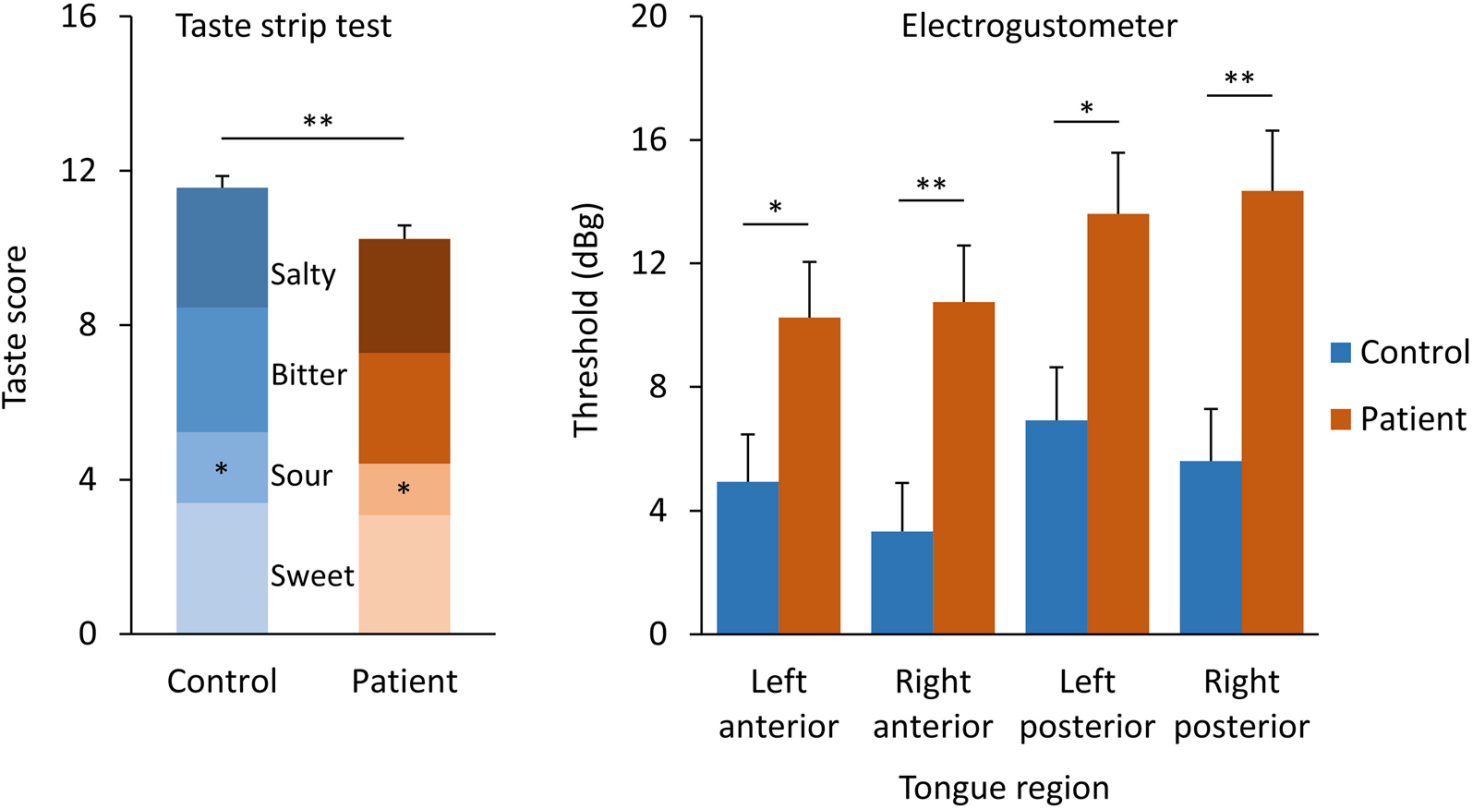
Mean value and staked SEM of the Taste strips score represented as sum of the single tastants, and of the electrogustometry threshold for the various tongue regions of controls and patients. *p < 0.05 while **p ≤ 0.01.

Patients and controls did not differ significantly in terms of taste spray scores. However, as expected, patients performed worse in the taste strips test compared to the control group (F_1,100_ = 8.34 *p* < 0.005). This difference was specific for sour, for which patients’ identification was lower than that of controls (F_1,103_ = 6.92, *p* = 0.010) (Fig. 1). In addition, patients had higher electrogustometry detection thresholds (decreased sensitivity) in all tongue quadrants compared to controls (F_1,103_ ≥ 4.74, *p* ≤ 0.032). Additionally, the self-rated taste function was evaluated significantly lower in patients than in controls (F_1,100_ = 81.1, *p* < 0.001).

### Time-domain results

**Figure 2:**
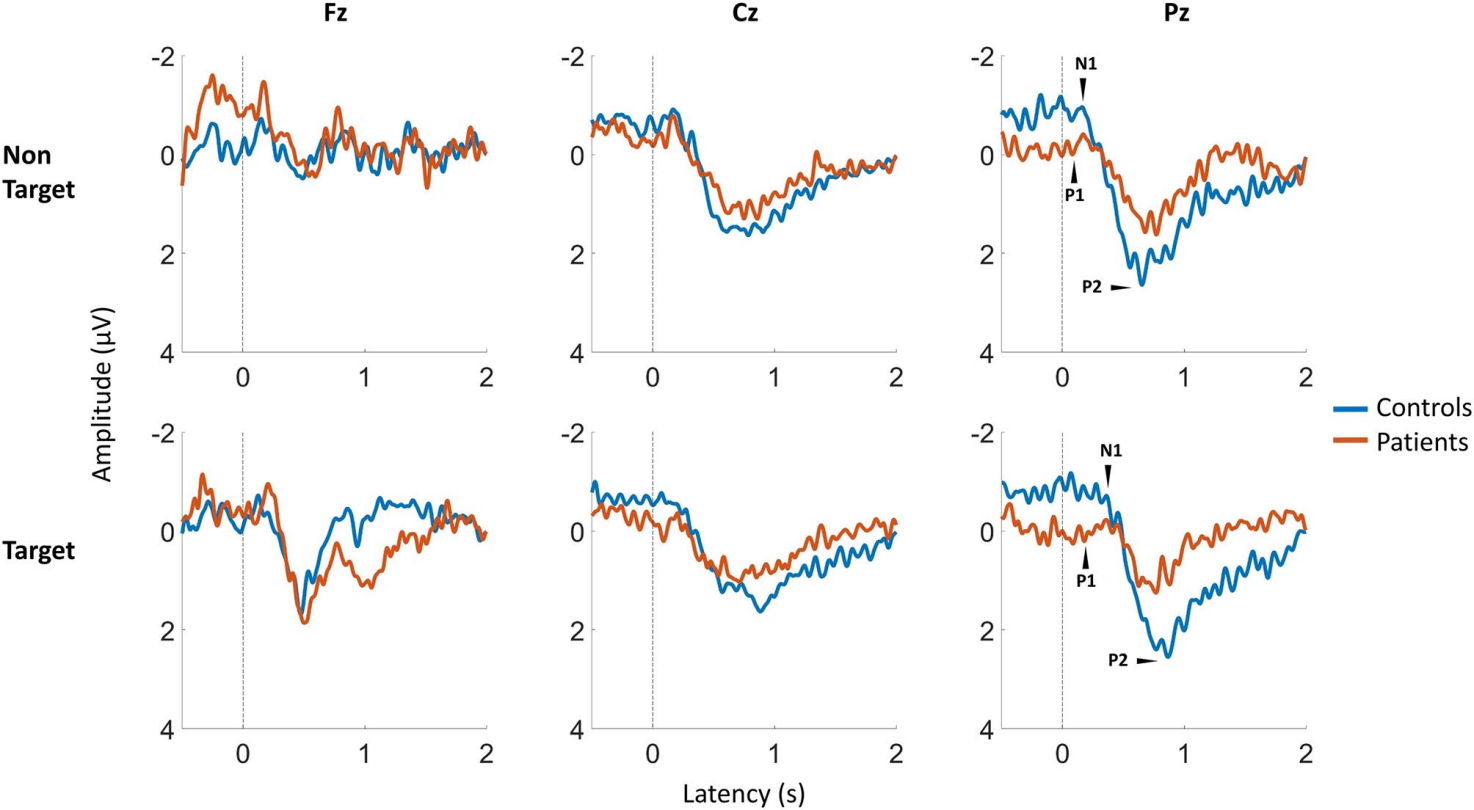
Grand average latencies and amplitudes of gustatory event-related potentials (gERPs) for controls and patients at recording positions Fz, Cz, and Pz. Stimulation started at 0 s (dashed lines). For visualization purposes, a band pass filter from 0.1 to 15Hz was used.

Results focused on three common reference electrodes for scalp position Fz, Cz, and Pz. Three peaks (P1, N1, and P2) were observed for control and patient groups after averaging the gERP recorded at electrodes Fz, Pz, and Cz: a small positivity after 0.2 s of stimulus onset (P1); a medium negative peak (N1) between 0.2 and 0.6 s; a large positivity (P2) between 0.4–1 s. Figure 2 shows averaged signals of gERP for controls and patients in midline electrodes, stratified for non-target and target stimulations (non-target solution: 1283.0 mM [75g NaCl/L distilled water], target solution: 171.0 mM [10g NaCl/L distilled water]).

**Figure 3:**
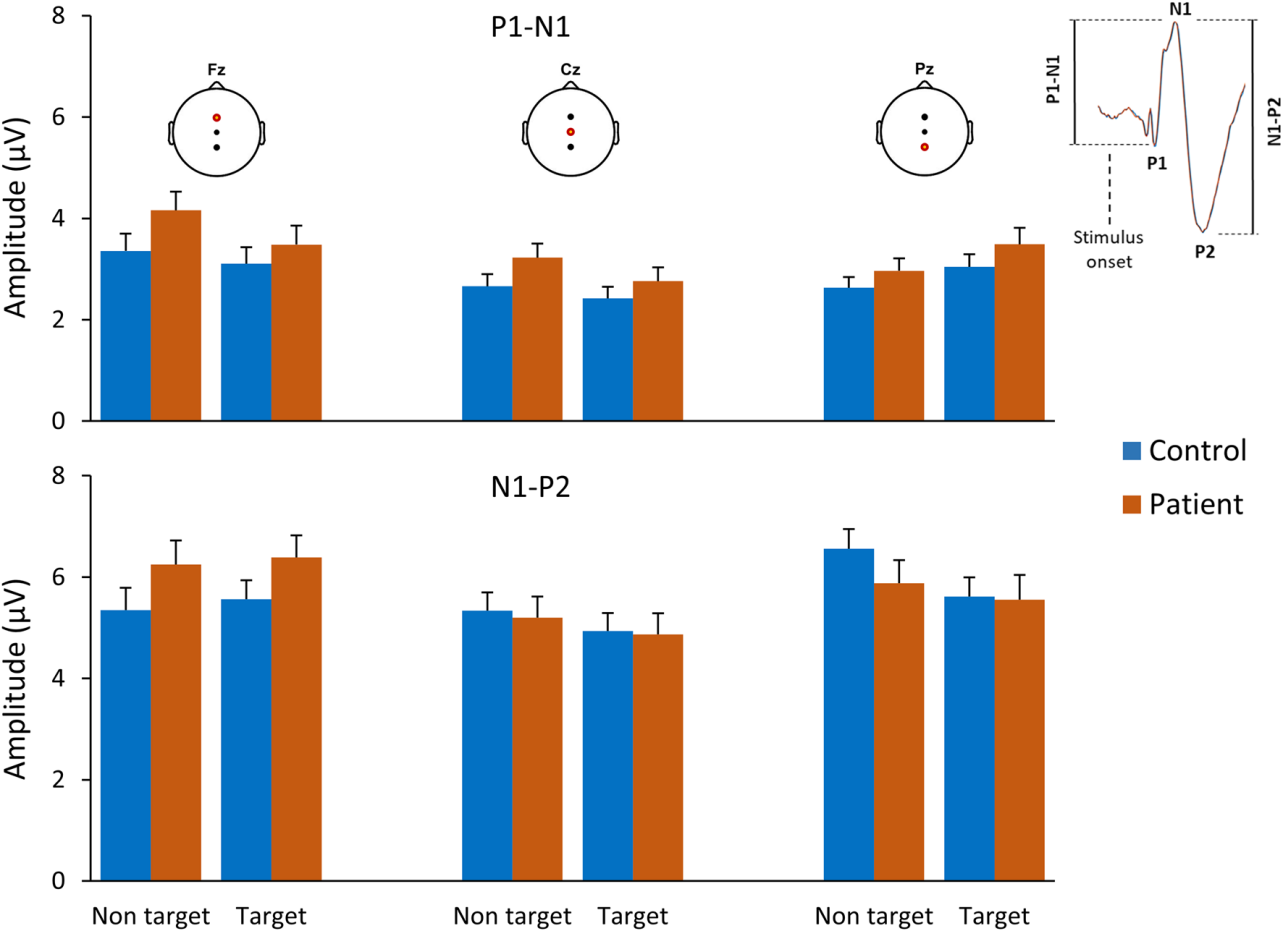
Mean values and SEM of peak-to-peak amplitudes for NaCl stimulations (75g/L non-target; 10 g/L target) in controls (*n* = 59) and patients (*n* = 44) for three recording sites (electrode positions Fz, Cz, and Pz).

Neither non target nor target stimuli evoked a significant difference in P1-N1 or N1-P2 amplitudes between heathy controls and patients. No differences in ERP peaks latencies were shown in different electrode positions (see Fig. 3). Responses elicited late in the recording showed a lower trend in amplitude and a longer latency, although lacking of significant differences.

### Time–frequency results

**Figure 4:**
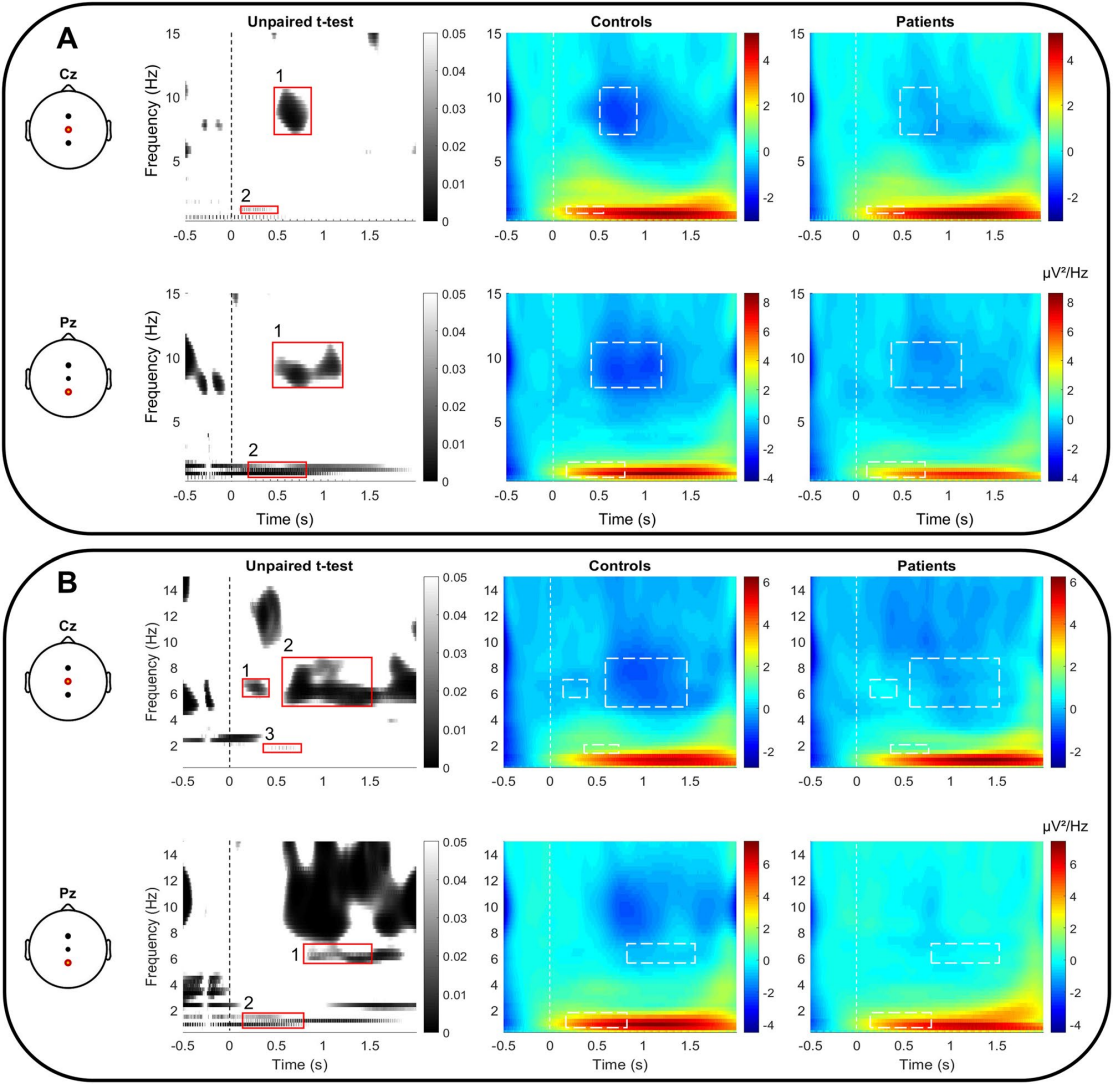
Time–frequency analysis (TFA) of the gERP responses to non-target (**A**) and target (**B**) stimuli in controls and patients for electrode scalp positions Cz and Pz. Black and white panels highlights area of the time–frequency maps that were significantly different between groups. Differences between groups are marked by red rectangles (unpaired *t*-test; p < 0.05) and numbered with a practical aim for further description. Maps represent group-level averaged frequencies across time, time-point 0 being the stimulus onset (vertical dashed lines). Color scales express the averaged power (µV^2^/Hz) and are normalized across groups. White-dashed rectangles reported in the map underline significantly different areas between groups.

Time frequency analyses revealed significant differences between controls and patients with taste dysfunction concerning brain responses to non-target and target stimuli (Fig. 4) recorded in Cz and Pz (unpaired *t*-test). Specifically, stimulations elicited a number of significant non phase-locked increases and decreases in EEG power with a stronger power in controls than in patients. In low frequencies (delta: 0.1–4 Hz), a long-lasting increase in amplitude was detected, which peaked at approximately 0.25 s after stimulus onset, showing a higher synchronization in controls. Stimulations elicited a significant long-lasting decrease of power in the higher frequency band oscillations (alpha: 8–12 Hz) starting approximately at 0.64 s after stimulus onset, which was higher in patients than in controls, for both recording sites (Cz and Pz). These differences were more pronounced after non-target (*p* ≤ 0.018) than after target stimulation (*p* ≤ 0.029). Based on these observations, distinct TFWs were defined and their information is summed in Table 1.

**Table 1:** Time–frequency windows in the frequencies between 0.1 – 12 Hz that were significantly different between groups.

Stimulus	Electrode	TFW	x limits (s)	Y limits (Hz)	T value	*p* value	xP min (s)	yP min (Hz)	Power (Controls/Patients)	Directionality
Non target	Cz	1	0.47 – 0.85	7.00 – 10.7	3.15	0.002	0.64	8.4	− 1.59/ − 0.49	HC < P (desynchronization)
		2	0.20 – 0.50	1.00 – 1.50	–2.36	0.018	0.26	1.2	3.22/2.64	HC > P (synchronization)
	Pz	1	0.44 – 1.22	7.60 – 11.0	2.92	0.004	0.70	8.7	− 1.96/ − 0.60	HC < P (desynchronization)
		2	0.20 – 0.80	0.65 – 1.75	–3.74	0.0002	0.21	0.9	4.40/3.29	HC > P (synchronization)
Target	Cz	1	0.20 – 0.42	5.50 – 7.10	2.62	0.009	0.30	6.3	− 0.06/0.57	HC < P (desynchronization)
		2	0.57 – 1.50	4.90 – 8.65	3.39	0.0007	0.77	6.3	− 0.68/0.13	HC < P (desynchronization)
		3	0.40 – 0.80	1.60 – 2.00	–2.17	0.030	0.57	1.8	1.79/1.15	HC > P (synchronization)
	Pz	1	0.80 – 1.50	5.50 – 6.80	2.76	0.006	1.39	6.0	− 0.06/0.16	HC < P (desynchronization)
		2	0.20 – 0.80	0.60 – 1.75	–2.89	0.004	0.22	0.9	4.11/3.50	HC > P (synchronization)

The lower *p* values of time–frequency windows (TFW) were extracted with corresponding T values, x and y (xP min and yP min), power value in each group and *t*-test directionality.

**Figure 5:**
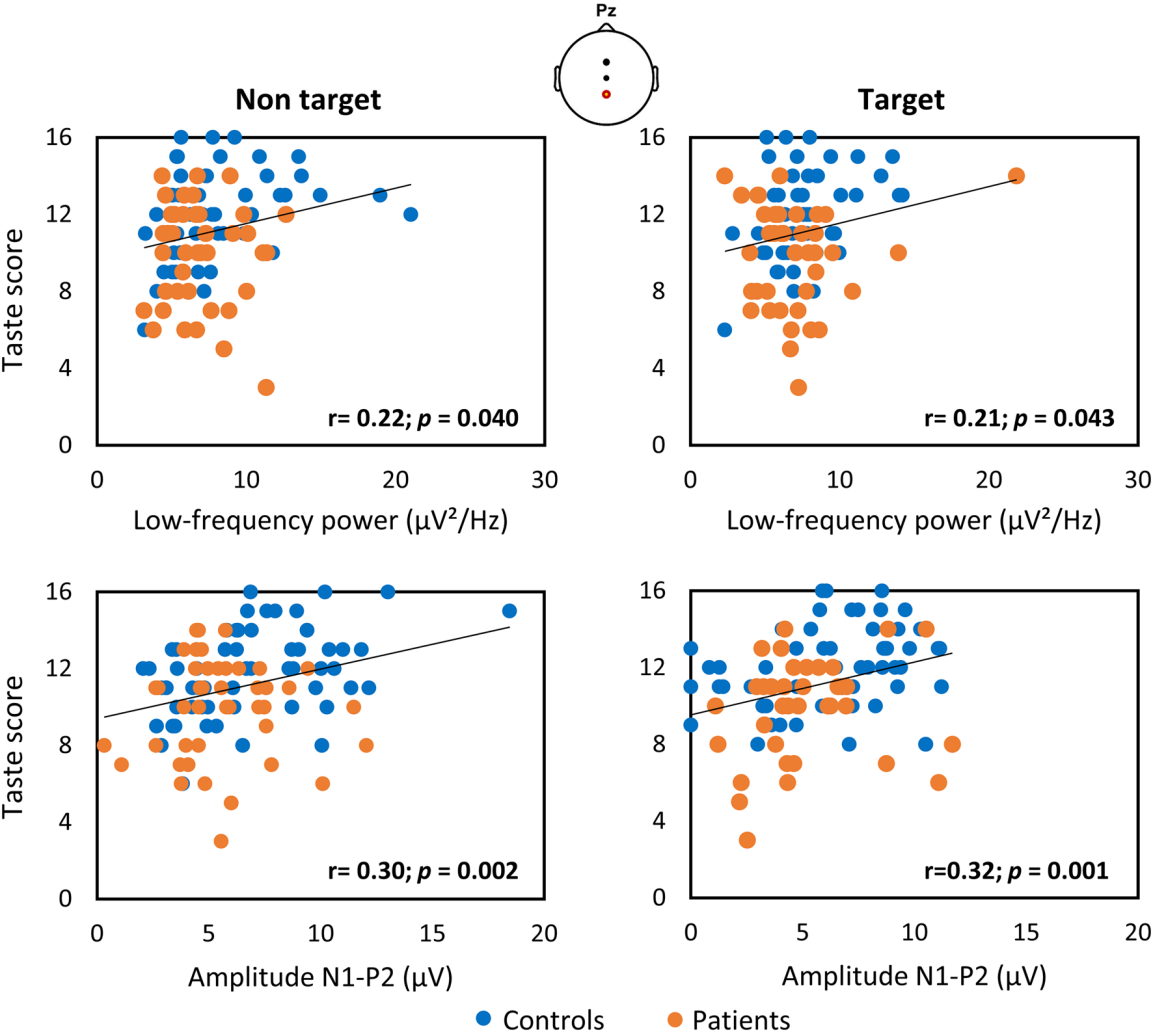
Correlations between psychophysical taste performance and power in low-frequency windows identified using across-trial averaging in the time–frequency domain, and amplitude of N1-P2 in Pz.

Finally, we examined the correlation between the psychophysical taste strip scores and the magnitude of the significant EEG measures identified in the time–frequency domain (Fig. 5). There was a significant positive correlation between taste scores and the maximum power recorded in the low-frequency TFW in Pz after stimulations (r ≥ 0.21; *p* = 0.043), and with N1-P2 amplitudes (r ≥ 0.30 *p* ≤ 0.002).

## Discussion

In the present study, we applied TFA on gustatory ERPs to reduce the signal noise that is present in traditional time-domain analysis of gERPs, and verify its use to discriminate healthy subjects from patients with dysgeusia. To this end, salty stimuli were provided with a computer-controlled gustometer. This onset was previously demonstrated to elicit gERPs^20,21,27,28^.

Analysis in the time–frequency domain revealed EEG activities that were not detected using conventional averaging. Following non-target stimulation, two distinctively different responses were identified in Cz and Pz. Healthy controls showed a higher neural synchronization in the low-frequency band, peaking approximately 0.25 s after stimulus onset, and a more pronounced stimulus-induced desynchronization in the high-frequency band, peaking approximately 0.70 s after stimulus onset, when compared to patients. Target stimulation elicited a similar pattern. These data confirm previous results showing that cortical representation of taste identity recognition recorded with EEG in healthy participants was encoded in the same delta-frequency range^26^, although their frequency map originated from the average of all electrodes.

Interestingly, taste information decoded with local field potential in rodents started increasing in the first second after stimulus presentation for frequency bands between 6 and 12 Hz^29^. Decreases or increases of power in selected frequency bands triggered by event-related phenomena are explained as the effect of decrease or increase in synchrony of neuronal populations, respectively^22^, which are recruited to process the gustatory input^30^. The delta-band synchronization elicited by the stimulation may reflect a stimulus-induced transient activation of neuronal populations involved in processing the salty input. Specifically, the significant differences in power found in the low-frequency range between groups suggest a reduced activation of central processing in patients with dysgeusia, for which the taste-induced synchronization and subsequent desynchronization involved a smaller neuronal network.

In this study, salty stimuli led to a desynchronization in the healthy group, which was more pronounced than in patients. To the best of our knowledge, only one study found a significantly lower band power between 8 and 12Hz at approximately 0.6s after stimulus onset^26^, but no previous studies analysed the non-phase locked desynchronization of neurons after gERPs. Since desynchronization was previously hypothesized to represent the deactivation of a neural population at a specific frequency band, one can speculate that the lower alpha band power found in healthy controls reflected the deactivation of the larger population of neurons that was previously activated after the gustatory stimulus. Other possible explanations are more speculative. First, in patients the neural population recruited might be more silent, or the desynchronization might have happened in different frequency band (i.e. beta or gamma, > 12Hz) that were not taken into consideration. Our hypothesis is supported in the literature by the desynchronization found in gamma frequencies after presentation of all taste qualities^26^. However, it should be validated analysing high-frequency desynchronization activities in dysgeusic patients. Second, the desynchronization might be delayed in time. It is worth mentioning that the patient group included different cases of dysgeusia, some of them mentioning long-lasting taste sensations. A late deactivation of the neuronal population may explain such long-lasting perceptions. Further studies are needed to confirm these hypotheses.

EEG average waveforms were analysed to measure three distinct peaks (P1, N1, and P2). To date, several studies have reported differences in ERP components after gustatory stimulation^18,27,31–33^. In the present study, the traditional analysis in the time-domain did not revealed any differences in peak-to-peak amplitudes between groups despite the different patterns that were evident in the grand average of groups of the ERPs signals in Fig. 2. Several factors might have contributed to decrease the signal-to-noise ratio. First, gustatory stimuli were delivered by means of an oddball paradigm, where the target stimulus was infrequently presented in a sequence of distracting non-target stimuli. The aim of this design was to strengthen the endogenous signal components, as a correlate of the cognitive processing of the stimulus in the EEG^34^. However, this led to an increase of repetitions number and a decrease of the Inter-stimulus interval (ISI) to 12s to avoid a lengthy procedure. Therefore, the short ISI and the higher number of non-target stimulations might have evoked habituation, while the length of the experimental procedure might have induced fatigue in subjects. Combining across-trial averaging in the time domain with the chosen oddball paradigm reduced changes in the EEG signal that were not time-locked to the stimulus onset. Consequently, the signal noise enhanced by averaging signals decreased the differences between groups^35,36^.

Overall, TFA significantly enhanced differences in stimulus-induced modulations of the amplitude of ongoing EEG oscillations. This enhancement was achieved by characterizing non phase-locked components that could not be identified using conventional time-domain averaging. These results are in accordance with previous studies that applied TFA on olfactory and trigeminal ERPs^23,37^, and the time frequency pattern of healthy controls are similar to spectral power estimates obtained after taste stimuli^26^. The lower power found in both low- and high-frequency TFWs seems to represent the lower gustatory sensitivity in patients with dysgeusia.

Given the correlation that previous studies found between psychophysical tests and the magnitude of chemosensory ERPs^23,38,39^, taste scores were correlated with the maximum power identified in low-frequency bands using across-trial averaging within subject in the time–frequency domain. The positive correlations strengthened the hypothesis that synchronization in delta band (0.1–4 Hz) reflects gustatory activation, which may be used as an index of taste-induced responses. The correlation supports the possible application of TFA in confirming the diagnosis of dysgeusia.

As expected, patients showed significantly reduced taste sensitivity in taste strips and electrogustometry testing, with threshold in patients being twice as high as in controls for all four regions of the tongue. Our results confirmed the strength of the electrogustometry analysis in detecting gustatory impairments^40,41^. In contrast, the whole mouth testing using supra-threshold taste sprays did not show any differences between the two groups. The reason for this may be that the majority of the tested patients (68%) were affected by parageusia and they consequently performed well in detection and identification of supra-threshold stimuli^42^.

In summary, the gERPs and synchronization and desynchronization values obtained using TFA appear to be a potential tool to study central gustatory responses, in both physiologic and pathologic states. TFA enhanced signal-to-noise ratio in the analysis of gustatory responses and reported additional differences between groups. The large number of subjects enrolled in the study, EEG-powers calculated by analysing the gustatory response in the time–frequency domain, were able to differentiate healthy controls from subjects with dysgeusia. The reason for this might be multiple: the oddball paradigm applied to focus on endogenous signal that limited the number of recordings. Further studies are necessary in order to additionally define the value of TFA on the gERP for the clinical usefulness.

## Conclusions

The present results suggest that taste activation assessed with gERPs and analyzed in the time and in the frequency domain produces different responses in patients with gustatory dysfunction and healthy controls. This is of diagnostic value for medical-legal reports or examination of non-cooperative patients, e.g. children. Future efforts will look at the prognostic value of gustatory parameters for patients with qualitative gustatory dysfunction. Despite the low prevalence of taste disorders, non-invasive electrophysiological diagnostic tools and prognostic instruments for taste disorders are needed for a better understanding of taste dysfunction and ultimately a better therapy, because taste dysfunction can have a strong influence on quality of life, dietary behaviour, social and mental health^43^.

## Methods

This study was conducted in accordance with the ethical principles of the declaration of Helsinki and was approved by the ethics committee of the medical faculty of the Technical University of Dresden (EK 286,112,008). All participants gave written consent of their participation in the study after being thoroughly informed about it. Subjects received moderate financial compensation.

### Participants

A total of 103 subjects participated in the study. Forty-four patients (35 women; 59.1 ± 2.4 years) were recruited at the outpatient Smell and Taste Clinic of the Department of Otorhinolaryngology of the Technische Universität Dresden presenting with dysgeusia. A detailed medical history was taken following a structured interview^44^. Their duration of symptoms as well as the cause of the taste disturbance are shown in Supplementary Table 1. At the same time, 59 healthy volunteers (34 females; 47.6 ± 2.1 years) with normal gustatory abilities over the age of 18 were enrolled in the study. Exclusion criteria were pregnancy, breastfeeding, significant health impairments (e.g. Diabetes mellitus, Parkinson's disease, renal insufficiency, oncological diseases, status after radiation therapy, medication history that can be associated with gustatory dysfunction), and alcohol or drug abuse.

### Procedure

Data acquisition was performed in one session and lasted approximately two and a half hours. At least 1 h prior to the start of the measurements, subjects were asked not to eat or drink anything but water. Recording of a standardized history was preceded by an otorhinolaryngological examination including nasal and oral endoscopy. Before starting the psychophysical testing of both smell and taste and then the electrophysiological examination, the study participants were asked to assess their own smell and taste function, respectively, on 9-point labelled scales, starting from “no perception” to “very good perception”. In addition, BDI-FS was used to screen for depression^45^.

#### Orthonasal olfactory assessment

In order to assess orthonasal olfactory function, the olfactory threshold (T score) was first determined with the help of odorized felt tip pens, the Sniffin ‘ Sticks (Burghart Messtechnik GmbH, Holm, Germany). Then the ability to discriminate and identify odors was tested (D and I score, respectively)^46^. The scores from the individual tests (each with a maximum of 16 points) were summed up to the TDI score, which allows age stratified classification into normosmia, hyposmia and anosmia^47^.

#### Gustatory assessment

##### Taste sprays

For screening of gustatory function taste sprays were applied to the tongue^43^. The subject’s task was to identify the four basic taste qualities (0.1 g/ml sucrose for “sweet”, 0.05 g/ml citric acid for “sour”, 0.0005 g/ml quinine hydrochloride for “bitter” and 0.075 g/ml sodium chloride for “salty” (Sigma-Aldrich Chemie GmbH, Munich, Germany)). The number of correctly identified tastants constituted the test score.

##### Taste strips

The taste strips test enables detection of thresholds for the above-mentioned basic taste qualities^8^. The strips are impregnated with various concentrations of the 4 basic tastes (sucrose – 0.4, 0.2, 0.1, 0.05 g/ml; citric acid – 0.3, 0.165, 0.09, 0.05 g/ml; quinine hydrochloride – 0.25, 0.1, 0.04, 0.016 g/ml; sodium chloride – 0.006, 0.0024, 0.0009, 0.0004 g/ml). A total of 16 taste strips—i.e. four different concentrations per taste—were presented in a semi-randomized order with overall increasing concentrations. After placing a strip on the tongue, participants were asked to determine the taste according to a "multiple forced choice" procedure. Thorough rinsing with water was used between taste presentations. The number of correctly identified tastants constituted the test score.

##### Electogustometry

Electrogustometry allows determining the detection threshold for an electrical impulse^48^. Stimuli were applied using an electrogustometer (Rion, TR-06, Sensonics Inc., Haddon Heights, NJ, USA; pulse length 500 ms; intensity between 4 μA and 400 μA ≅ -6 gustatory decibel gdB to 34 gdB).

The monopolar (anodic) stimulation was performed in four different areas: in the left and right anterior part of the tongue, supplied by the chorda tympani (facial nerve), and in the left and right posterior third of the tongue innervated by the glossopharyngeal nerve^9,49^. The “two alternative forced choice test” was used to determine the threshold. Starting with the lowest intensity level, the tongue was touched twice per cycle, with stimulation only occurring once. Participants then indicated when the stimulus was present. If the answer was wrong, the stimulus intensity was increased until two correct answers were given sequentially. Then the stimulus intensity was reduced again until the next incorrect answer occurred. The average of the last four of seven of these turning points were used as a threshold estimate.

#### gERP measurement

To generate gERP, participants received various taste stimuli using a gustometer while simultaneously recording EEG. Participants were comfortably seated with their extended tongue in front of the gustometer outlet. The gustometer GU001 (Burghart Messtechnik GmbH, Wedel, Germany) was used to present warm solutions (36 °C at outlet of gustometer) to the subject with predefined computer-controlled pulse duration and inter-stimulus interval (ISI). This creates clearly defined, reproducible taste stimuli on the tongue^21,50^. The entire measurement was divided into three equal-length sections, between which the study participants had the opportunity to relax and drink water. In total, the recording lasted approximatively 35 min.

Two salty solutions were used as stimuli, which differed only by the concentration of sodium chloride (NaCl, CAS number: 7647–14-5, Sigma-Aldrich Chemie GmbH, Munich, Germany). A liquid mimicking human saliva (potassium chloride 25 mM, sodium hydrogen carbonate 2.5 mM), was released continuously in between the stimuli^51^. The sequence of 95 non-target and 25 target stimuli was sprayed onto the participants’ tongue (pulse volume 100 μl for 500 ms) in a randomized order according to an oddball paradigm (Squires et al. 1975)^34^. The ISI was 12 s in which the study participant was permanently exposed to a saliva replacement solution in order to minimize artefacts of pressure and temperature changes, and to constantly moisten the tongue surface. This technique allows the recording of pure gustatory stimulus response^51^. Throughout the EEG recording, participants received white noise (approximately 50 dB) through headphones to mask switching clicks of the stimulation device; they performed a tracking task on a computer screen in order to maintain vigilance and to reduce unwanted eye movements; they were asked to count the number of target stimuli in order to focus the participants' attention.

##### EEG recording and analysis

EEG was recorded with an electrode cap (BioSemi-CAP) from 128 Ag–AgCl active electrodes (BioSemi, Amsterdam, Netherlands) using the international 10–20 system. The signals from each electrode were amplified using the BioSemi Active Two AD-box. Two Ag–AgCl flat-type electrodes above the lateral extremity of the eyebrows and two on the lateral side of the eyes were applied to identify eye-blinks in the signal. The sampling frequency was 512 Hz (BioSemi, ActiveView 605 software).

All EEG processing was performed with the Letswave 7 software (Nocions, Brussels, Belgium) and EEGLAB 2020 (La Jolla, CA, USA). Recordings were analyzed for segments of 2500 ms (500 ms pre-trigger baseline) and filtered with a band-pass filter of 0.3–30.0 Hz. Movement and blinking artifacts were identified and removed using Independent Component Analysis (ICA; EEGLAB 2020, La Jolla, CA, USA). Finally, for each study participant and each derived channel, an averaged signal for the target and non-target stimuli was obtained, the components of which were heuristically measured with regard to their peak latencies (ms) and amplitudes (µV) and reported as a complex (N1–P2). Within the same subject, the number of target and non-target epochs were equalized in a random manner. Literature of gustatory responses usually reports midline positions as the best recording site^20,21,27^. To focus the data analysis, only recordings from midline electrodes Fz, Cz, and Pz were included in this analysis. A time–frequency analysis (TFA) based on the continuous Morlet Wavelet Transform (CWT) of individual EEG epochs was used to characterize the amplitude of oscillatory activity as a function of time and frequency, according to previous studies^52,53^. Based on previous findings in olfactory ERPs^25^, frequencies ranging from 0.5 to 12 Hz were taken into consideration.

### Statistical analysis

One-way ANCOVA controlling for age was used to compare results between groups, and chi-square analysis was applied to compare frequencies in gender. Differences in amplitude and latency of gERP peaks between groups were evaluated using repeated measures ANCOVA with peaks as repeated measure factors, subjects’ group as between factor, and age as covariate. If a significant effect was present, post hoc comparisons were performed using Bonferroni correction. SPSS® Version 28 (Statistical Packages for Social Sciences Inc., Chicago, Ill., USA) was used for statistical analyses. Data are shown as means ± standard error (SEM), and effect sizes are reported as Eta squared values (η^2^).

To assess significant differences in increases and decreases of signal amplitude observed in the group-level average time–frequency maps, a one-sample *t*-test between groups was performed using the amplitudes estimated in each subject. This produced, for each type of stimulus, a time–frequency map highlighting the regions where the EEG signal deviated significantly from each group (*p* < 0.05). These statistical maps were then used to define a number of time–frequency windows (TFW), circumscribing stimulus-induced EEG responses. Maximum or minimum power values (µV^2^/Hz) within each TFW were calculated in the averaged map as summary values estimating response magnitude, while the ones calculated using across-trial averaging within the same subject were used as index of magnitude to correlate them with psychophysical taste test and different measures of the gERPs using Pearson’s correlation.

The level of significance was set at *p* < 0.05. Matlab software (Version R2018b, The MathWorks, Natick, Mass., USA) and the associated toolbox Letswave were used to plot the graphs.

### Supplementary Information

Supplementary Information.

## Data Availability

The datasets generated and/or analyzed during the current study are available from the corresponding author upon reasonable request.
