# Supplementary material for: Time–frequency analysis of gustatory event related potentials (gERP) in taste disorders
Source: Sci Rep. 2024 Jan 30;14:2512. doi: 10.1038/s41598-024-52986-5 (PMC10827706; doi:10.1038/s41598-024-52986-5)
Supplement: Supplementary file 1 — Supplementary Information. [file 41598_2024_52986_MOESM1_ESM.docx]

**Table S1.** Frequency and types of dysgeusia, cause, therapy, and co-olfactory dysfunction

| **Description** | | **Frequency** | **Percent** |
| --- | --- | --- | --- |
|  | Gender  male/female | 9/35 | 20.5% / 79.5% |
|  | Smoking | 9 | 20.5% |
| Type of Dysgeusia | Salty | 16 | 36.4% |
|  | Bitter | 10 | 22.7% |
|  | Sweet | 1 | 2.3% |
|  | Sour | 1 | 2.3% |
|  | Hypogeusia | 11 | 25.0% |
|  | Other | 5 | 11.4% |
| Duration of illness | < 3 months | 8 | 18.2% |
|  | < 1 year | 19 | 43.2% |
|  | > 1 year | 16 | 36.4% |
| Cause | Idiopathic | 18 | 40.9% |
|  | Iatrogenic | 12 | 27.3% |
|  | Post infection | 8 | 18.2% |
|  | Drug-Induced | 4 | 9.1% |
|  | Other | 2 | 4.5% |
| Therapy | Zinc | 33 | 75.0% |
|  | Coldastop | 4 | 9.1% |
|  | Alpha-Lipoic acid | 2 | 4.5% |
|  | Acupuncture | 2 | 4.5% |
|  | No treatment | 3 | 6.8% |
| Olfactory dysfunction | Chronic nasal problems | 10 | 22.7% |
|  | Hyposmia | 8 | 18.2% |
|  | Functional Anosmia | 6 | 13.6% |
|  | Parosmia | 1 | 2.3% |
|  | Phantosmia | 3 | 6.8% |

Type of Dysgeusia was assessed by chemosensory tests (Taste Strips test)
